# Diagnostic and Therapeutic Approaches in Periodontology: From Traditional Concepts to Modern Innovations

**DOI:** 10.3390/biomedicines14091916

**Published:** 2026-08-26

**Authors:** Tatiana Chacón, Óscar Zuluaga-López, Gloria María Sandoval-Llanos, Maria Camila Piedrahita Posada, Brenda Yuliana Herrera-Serna

**Affiliations:** Oral Health Department, Universidad Autónoma de Manizales, Manizales 17001, Colombia; tatianacha@autonoma.edu.co (T.C.); ohzuluaga@autonoma.edu.co (Ó.Z.-L.); gsandoval@autonoma.edu.co (G.M.S.-L.); maria.piedrahitap@autonoma.edu.co (M.C.P.P.)

**Keywords:** periodontitis, biomarkers, precision medicine

## Abstract

**Objectives:** To synthesize current evidence regarding advances in periodontal diagnosis and therapy, with emphasis on molecular biomarkers, omics technologies, microbiome profiling, digital imaging, and artificial intelligence-based analytical models that support the transition toward precision periodontology. **Methods:** This narrative review examines contemporary evidence on emerging molecular, microbiological, and digital technologies applied to periodontal diagnosis, prognostic assessment, and therapeutic planning. The review includes studies addressing salivary and gingival crevicular fluid biomarkers, microbiome characterization, omics approaches, cone-beam computed tomography, three-dimensional imaging, machine-learning algorithms, and personalized periodontal therapies. Relevant literature was identified through searches in major biomedical databases, including PubMed/MEDLINE, Scopus, and Web of Science, focusing on studies published on periodontal diagnostics, biomarkers, digital technologies, artificial intelligence, and precision medicine approaches in periodontology. **Results:** Peer-reviewed articles addressing innovative diagnostic and therapeutic approaches in periodontology were considered. Priority was given to studies evaluating clinical applicability, diagnostic performance, prognostic utility, and personalized treatment strategies integrating molecular and digital technologies. **Conclusions:** Emerging molecular and digital technologies are reshaping periodontal diagnosis and therapy by improving disease detection, risk prediction, and individualized treatment planning. Biomarkers, omics technologies, microbiome profiling, and artificial intelligence-assisted imaging may enhance diagnostic precision and clinical decision-making. These developments support the implementation of precision periodontology; however, challenges related to biomarker validation, algorithm standardization, cost, and accessibility remain barriers to routine clinical adoption. Further research is necessary to validate these approaches and facilitate their integration into periodontal practice. The integration of biomarkers, omics technologies, advanced imaging, and artificial intelligence may improve early periodontal diagnosis, prognostic assessment, and personalized treatment planning. These innovations support the transition toward precision periodontology and have the potential to enhance clinical decision-making, treatment outcomes, and long-term periodontal health in routine dental practice.

## 1. Introduction

Periodontitis is a highly prevalent chronic inflammatory disease that represents a major burden for both oral and systemic health. It is characterized by a dysbiotic subgingival microbiome and a dysregulated host immune response, leading to progressive destruction of the tooth-supporting tissues. In routine practice, diagnosis still relies mainly on clinical parameters and two-dimensional radiographic assessment. Although these approaches remain indispensable, they largely reflect cumulative tissue damage and provide limited insight into ongoing disease activity or future progression.

Recent advances in molecular biology and digital technologies have substantially reshaped the current understanding of periodontitis as a complex and multifactorial condition. The identification of biomarkers in saliva and gingival crevicular fluid, together with the development of omics-based approaches and microbiome profiling, has enabled a more detailed characterization of host–microbe interactions. At the same time, progress in digital imaging and artificial intelligence has improved the potential for more accurate detection, classification, and risk assessment.

However, the clinical translation of these innovations remains uneven. Key challenges include the lack of standardized and validated biomarkers, limited external validation of emerging technologies, and the practical difficulties of integrating molecular and digital data into routine clinical workflows. As a result, their impact on everyday periodontal practice is still evolving.

## 2. Materials and Methods

### 2.1. Study Design and Reporting Framework

This work was conceived as a narrative review. Because narrative reviews are not bound by the procedural requirements that govern systematic reviews, its preparation and reporting were guided by the Scale for the Assessment of Narrative Review Articles (SANRA), which structures a narrative review around the justification of its importance, the statement of its aims, the description of the literature search, referencing, scientific reasoning, and the presentation of relevant data [1]. In line with this design, no formal systematic screening, quantitative risk-of-bias scoring, or meta-analysis was performed; instead, the retrieved evidence was appraised and integrated qualitatively. The methodological transparency described below is intended to make the selection and synthesis of the evidence reproducible and to allow readers to judge the strength of the sources on which the review is based.

### 2.2. Data Sources and Search Strategy

A structured literature search was conducted in three major biomedical databases—PubMed/MEDLINE, Scopus, and Web of Science—covering the period from January 2015 to December 2025. The search was last updated on 20 January 2026. To reduce the risk of omitting relevant work, the electronic search was complemented by manual screening (“snowballing”) of the reference lists of the retrieved articles and of pertinent review papers, allowing the identification of additional sources not captured by the database queries.

The search strategy combined free-text keywords with Medical Subject Headings (MeSH), linked through the Boolean operators AND and OR. The core terms included “periodontitis,” “periodontal diagnosis,” “periodontal therapy,” “biomarkers,” “gingival crevicular fluid,” “saliva,” “omics,” “microbiome,” “cone-beam computed tomography,” “digital imaging,” “artificial intelligence,” “machine learning,” “host-modulation therapy,” “regenerative therapy,” and “precision medicine.” Representative search strings included: (“periodontitis” AND (“biomarkers” OR “omics” OR “microbiome”)); (“periodontitis” AND (“digital imaging” OR “cone-beam computed tomography” OR “artificial intelligence” OR “machine learning”)); and (“periodontitis” AND (“host-modulation therapy” OR “regenerative therapy” OR “precision medicine”)).

### 2.3. Eligibility Criteria

Studies were considered eligible when they (i) were published in English between 2015 and 2025; (ii) addressed diagnostic or therapeutic approaches in periodontology, with particular emphasis on molecular, digital, or computational innovations; and (iii) corresponded to original research articles (in vitro, in vivo, observational, or interventional), systematic reviews, or authoritative narrative reviews and consensus or classification reports. Seminal earlier publications were retained only when they were essential for conceptual or historical context, most notably the 2017 World Workshop classification of periodontal and peri-implant diseases and conditions.

Records were excluded when they (i) were conference abstracts, editorials, letters, or unpublished and grey-literature material; (ii) were not available in full text; (iii) were written in languages other than English; or (iv) were unrelated to the central focus of the review or provided only redundant information already covered by sources of higher methodological quality.

### 2.4. Study Selection

Titles and abstracts retrieved from the databases were first screened for relevance against the eligibility criteria, and potentially relevant records were subsequently assessed in full text. Screening and selection were carried out by the authors; disagreements regarding inclusion were resolved through discussion until consensus was reached. When several sources reported on the same topic, priority was given to the most recent and methodologically robust evidence in order to keep the synthesis current and to limit redundancy.

### 2.5. Quality Appraisal and Evidence Synthesis

Consistent with the narrative nature of the review, the methodological quality of the included studies was appraised qualitatively rather than through a standardized scoring instrument. For each source, the authors considered the study design, the sample size, the diagnostic or classification criteria applied, the robustness of validation (particularly for biomarker- and artificial-intelligence-based studies, where external validation is frequently limited), and potential sources of bias or confounding. Greater interpretive weight was accordingly given to studies with stronger designs, adequate sample sizes, and independent or external validation.

The extracted information was organized thematically and synthesized narratively around the main domains of the review: conventional periodontal diagnosis; molecular and omics-based diagnosis; microbiome profiling; advanced digital and imaging-based diagnosis; predictive and machine-learning models; and modern, diagnosis-guided therapeutic strategies. This thematic structure was chosen to integrate clinical, molecular, and digital evidence and to support a coherent transition from traditional concepts toward precision periodontology.

The database searches retrieved a combined total of 254 records (131 in PubMed/MEDLINE, 112 in Scopus, and 11 in Web of Science). After removal of 76 duplicates, 178 records were screened by title and abstract, 62 were assessed in full text, and 4 additional sources were identified through snowballing, yielding a final set of 66 references included in the qualitative synthesis.

## 3. Periodontal Diagnosis

### 3.1. Clinical Parameters and Staging–Grading Classification

The periodontal clinical examination remains the reference standard for diagnosing periodontitis, determining its extent and severity, and assessing therapeutic outcomes. Probing depth (PD), clinical attachment level (CAL), and bleeding on probing (BOP) provide complementary rather than interchangeable information. PD reflects pocket anatomy and inflammatory changes but is influenced by probing force, tissue condition, probe angulation, and gingival-margin position. CAL more directly represents cumulative attachment loss, although it cannot distinguish historical destruction from current disease activity [2,3,4]. Measurement reproducibility also depends on examiner calibration and probe design. Pressure-sensitive and electronic probes may improve intra- and inter-examiner consistency, but current evidence does not demonstrate their general diagnostic superiority over manual probing; therefore, manual probing remains the clinical standard [5].

BOP is a sensitive indicator of gingival inflammation, but a positive finding has limited value for predicting future attachment loss. Its absence is more informative for periodontal stability than its presence is for identifying a site that will progress. Furcation involvement, tooth mobility, and periodontitis-related tooth loss similarly provide essential information on accumulated damage, prognosis, and treatment complexity, but they are not direct measures of ongoing biological activity [2,4]. Consequently, no isolated clinical variable defines active periodontitis. Diagnosis requires integration of inflammatory findings, attachment loss, anatomical complexity, longitudinal changes, and relevant systemic and behavioral risk factors.

The 2017 World Workshop classification organizes these findings through staging and grading. Staging summarizes severity and management complexity using interproximal CAL, radiographic bone loss, periodontitis-related tooth loss, deep pockets, vertical defects, furcation involvement, and functional impairment. Stage I corresponds primarily to 1–2 mm of interproximal CAL and Stage II to 3–4 mm, whereas Stages III and IV involve CAL of at least 5 mm and are differentiated by tooth loss, anatomical complexity, and functional consequences. Stage III may include the loss of up to four teeth because of periodontitis, while Stage IV includes the loss of five or more teeth and may involve masticatory dysfunction, occlusal instability, or the need for complex rehabilitation [6,7]. Staging therefore describes both the burden of previous destruction and the clinical demands of treatment; it should not be interpreted as a measure of current inflammatory activity.

Grading estimates the probable rate of progression. Direct longitudinal evidence is preferred, but when previous records are unavailable, grade assignment relies mainly on the radiographic bone-loss-to-age ratio and is modified by smoking and diabetes [6,7]. This introduces a risk-oriented dimension into case definition, but the estimate remains indirect and partly dependent on the quality of radiographs, the attribution of tooth loss, and the availability of reliable medical and behavioral information. Thus, grading should guide risk communication, monitoring intensity, and risk-factor control rather than be treated as an independently validated prediction of future attachment loss.

Implementation is also influenced by examiner training and interpretation of borderline cases. Recent evidence indicates moderate inter-examiner reliability and moderate-to-very-good intra-examiner reliability, while a multinational survey of 1113 professionals found that 78% used the classification and identified complexity as its most frequent barrier [8]. The framework nevertheless provides the most coherent current system for integrating severity, complexity, and estimated progression. Its clinical value is maximized by standardized data collection, examiner calibration, transparent documentation of grade modifiers, and longitudinal reassessment [9].

Conventional clinical parameters therefore remain the global standard for diagnosis and outcome assessment. Their principal limitation is temporal: most describe inflammation observed at examination or tissue destruction that has already occurred. Molecular biomarkers, quantitative imaging, and predictive models may eventually add information on biological activity or future risk, but their incremental value must be demonstrated against—not instead of—comprehensive clinical and radiographic assessment [7,9].

### 3.2. Two-Dimensional Radiographic Assessment

Radiographic examination complements clinical probing by depicting mineralized periodontal structures. Panoramic, periapical, and bitewing radiographs support evaluation of alveolar bone levels, the distribution and pattern of bone loss, root morphology, furcation areas, and local contributing factors. Radiographic bone loss is central to staging, and the bone-loss-to-age ratio contributes to grading when direct evidence of progression is unavailable [6,10]. Nevertheless, radiographs show the structural consequences of disease rather than current inflammatory or microbial activity and must be interpreted together with PD, CAL, BOP, mobility, and longitudinal clinical information [2,11].

Panoramic radiography provides broad anatomical coverage and is useful for an initial overview of the dentition, generalized bone loss, missing teeth, and findings requiring targeted investigation. Its unequal magnification, superimposition, positioning sensitivity, and lower spatial resolution limit precise site-level measurement. Periapical and bitewing radiographs provide greater detail of interproximal bone and, when acquired with standardized positioning and projection geometry, are better suited to longitudinal comparison. Bitewings are particularly useful for posterior crestal bone levels, whereas periapical images provide additional information on roots, the periodontal ligament space, lamina dura, periapical tissues, and local anatomy [11]. Accordingly, panoramic and intraoral imaging serve complementary purposes rather than representing interchangeable alternatives.

All two-dimensional modalities compress three-dimensional anatomy into a single plane. Buccal and lingual defects, dehiscences, fenestrations, osseous craters, and complex furcation or intrabony morphology may therefore be underestimated or obscured. Apparent bone levels are also affected by receptor position, beam angulation, exposure, contrast, image processing, patient movement, anatomical reference points, and observer experience. Digital enhancement can facilitate visualization and measurement but cannot recover information lost through projection or positioning errors [11]. Standardized acquisition is consequently essential when images are compared over time.

Despite these limitations, two-dimensional intraoral radiography remains the preferred imaging approach for most periodontal diagnosis and follow-up because it is accessible, relatively inexpensive, involves limited radiation exposure, and has an established interpretive framework. Three-dimensional imaging should be reserved for selected situations in which the expected diagnostic or treatment-planning benefit justifies the additional radiation and cost. Neither two- nor three-dimensional imaging can independently identify active attachment loss; the clinically relevant question is whether the examination provides information that changes diagnosis or management beyond conventional assessment [9,11].

### 3.3. Culture-Based Periodontal Diagnosis

Anaerobic culture was fundamental to the identification and phenotypic characterization of microorganisms associated with destructive periodontal conditions. It requires subgingival sampling, transport under conditions that preserve anaerobes, inoculation on selective or non-selective media, incubation, and identification of recovered colonies. Although current disease models emphasize polymicrobial dysbiosis and host–microbiome interactions, culture remains distinct from DNA-based methods because it demonstrates recoverable microbial viability and permits phenotypic antimicrobial-susceptibility testing [12,13,14].

Its diagnostic scope is nevertheless limited. Recovery depends on the sampled site, transport conditions, media, incubation, and the ability of fastidious microorganisms to grow under laboratory conditions. Culture therefore represents the organisms recoverable under the selected conditions rather than the complete in vivo community. It may miss uncultivated or low-abundance taxa and does not characterize community interactions, virulence expression, or the host inflammatory response. Conversely, recovery of *Aggregatibacter actinomycetemcomitans*, *Porphyromonas gingivalis*, *Prevotella intermedia*, or *Tannerella forsythia* does not demonstrate that the organism is causing active destruction at the sampled site [13,15].

Culture has consequently shifted from routine etiological diagnosis toward selected research and clinical applications, particularly viability assessment, isolate characterization, antimicrobial-resistance surveillance, and susceptibility testing when adjunctive antibiotics are being considered. A synthesis of culture-based studies documented post-treatment reductions in several disease-associated taxa, but heterogeneous sampling and culture protocols limit comparisons across studies [16]. More broadly, recent evidence does not show that culture or another microbial test can reliably identify active attachment loss, predict progression after treatment, or improve outcomes when used to direct therapy [9,15]. Culture therefore retains a specific microbiological function but should not be interpreted as a standalone periodontal diagnostic test.

### 3.4. Targeted Molecular Diagnostic Tests

Targeted molecular tests shifted periodontal microbiology from phenotype-based identification toward detection of predefined genetic or enzymatic targets. They can identify selected microorganisms without cultivation and, in some formats, estimate their abundance. However, periodontitis is a dysbiosis-associated inflammatory disease whose expression depends on community structure, strain-level properties, the local environment, and host susceptibility. Detection of an individual taxon therefore provides etiological information but does not establish current disease activity, causal primacy at a specific site, or future attachment loss [12,13,17].

#### 3.4.1. Analytical Characteristics of Targeted Tests

Checkerboard DNA–DNA hybridization enabled simultaneous screening of multiple predefined bacterial taxa and contributed to the characterization of subgingival microbial complexes. Its closed design, however, restricts detection to organisms represented by suitable probes, and performance depends on probe quality, hybridization conditions, and standards. The BANA test detects trypsin-like enzymatic activity associated principally with *P. gingivalis*, *Treponema denticola*, and *T. forsythia*. Although rapid and inexpensive, it neither identifies the organism responsible for a positive reaction nor characterizes the broader community. Both methods are consequently more relevant historically or for predefined screening than as contemporary diagnostic standards [13,15,18].

Polymerase chain reaction (PCR) detects small quantities of pathogen-specific DNA or selected 16S rRNA gene regions without prior cultivation. Quantitative real-time PCR (qPCR) additionally estimates target abundance, while multiplex qPCR can assess several predetermined species in the same reaction. Experimental comparisons have demonstrated greater analytical sensitivity and faster quantification than viable plate counting, but analytical performance in concentrated bacterial suspensions does not establish clinical validity [19]. Clinical studies have shown technical agreement between in-house, commercial, or chairside assays and laboratory qPCR; however, small samples, absence of healthy or clinically representative comparison groups, higher detection limits in chairside formats, and lack of independent validation restrict conclusions about diagnostic discrimination or prognosis [20,21].

PCR-based results also depend on primer and probe design, amplification thresholds, sample collection, bacterial strain diversity, contamination control, and the range of predefined targets. Conventional qPCR amplifies DNA from viable and non-viable organisms; lower bacterial counts after propidium monoazide pretreatment illustrate how conventional assays may overestimate viable burden [22]. Thus, PCR and qPCR provide high analytical sensitivity and targeted quantification but cannot independently characterize community-wide dysbiosis, microbial viability, active tissue destruction, or future progression [13,22].

#### 3.4.2. Evidence and Clinical Utility

Four evidentiary levels should be distinguished when evaluating targeted microbial tests: analytical validity, cross-sectional diagnostic accuracy, prognostic validity, and clinical utility. Reliable detection of a target does not prove that the result distinguishes disease from health; discrimination of established cases does not demonstrate prediction of progression; and prognostic association does not establish that using the test improves treatment decisions or patient outcomes. Much of the periodontal literature supports the first two levels, whereas evidence for prognosis and clinical utility remains limited [9,13,15].

Cross-sectional studies show that selected taxa and microbial combinations can distinguish clinically established periodontitis from health under controlled conditions. A meta-analysis reported pooled sensitivity of 84.2%, specificity of 85.4%, and an area under the curve (AUC) of 0.864 for *P. gingivalis*, with some combinations outperforming individual taxa [23]. These estimates, however, predominantly reflect classification of participants already separated by clinical or radiographic criteria. Case–control comparisons of clearly diseased and healthy groups may exaggerate performance relative to populations containing gingivitis, early or treated periodontitis, and relevant systemic or behavioral modifiers. Differences in case definitions, sample types, sampled sites, platforms, targets, and thresholds further limit generalizability [15,23].

Prognostic evidence is substantially weaker. In the systematic review by Bostanci et al., longitudinal studies of microbial prediction showed high or unclear risk of bias and inconsistent predictive values. *A. actinomycetemcomitans* showed no meaningful discrimination in one adult model, while *P. gingivalis* generally provided only moderate prognostic performance. Studies of post-treatment outcomes were few, small, and heterogeneous in treatment, sampling time, microbial endpoint, and definition of clinical resolution [15]. The 20th European Workshop therefore concluded that no microbial marker currently has acceptable clinical utility for predicting periodontitis progression after therapy or for establishing clinically applicable thresholds [9].

Evidence for test-guided treatment is also inconclusive. In a retrospective cohort of 425 patients, systemic antibiotics were prescribed according to detection of *A. actinomycetemcomitans*, and a larger proportion of antibiotic-treated patients achieved the study endpoint. Because microbial status determined allocation and no randomized comparison of alternative test-guided strategies was performed, the study cannot show that testing caused the difference in outcome [24]. A second report from the same population found only 63% agreement between microbiology-based and age/severity-based antibiotic indications, but it did not establish the superiority of either strategy and does not represent independent validation [24,25].

Models combining microbial and host-derived information may be more biologically plausible than isolated pathogen detection. In a six-month longitudinal study of 114 patients, models incorporating plaque levels of *P. gingivalis* and *T. forsythia* with salivary and serum inflammatory markers identified progressors more sensitively than individual variables. Nevertheless, 27 candidate biomarkers were considered, progression occurred in only 28 participants, and performance was evaluated within the study population. External validation, calibration, and comparison with a clinical-only model are therefore required before implementation [26].

Targeted microbial tests may provide complementary information in selected cases, particularly when a specific microbial question is clinically justified. They should not replace clinical and radiographic examination or independently determine antimicrobial therapy. Translation requires standardized sampling and laboratory procedures, prespecified thresholds, representative populations, blinded evaluation, external multicenter validation, and prospective studies showing incremental value, improved decisions, patient-relevant benefit, or acceptable cost-effectiveness [9,15].

### 3.5. Omics and Susceptibility Biomarkers

Omics technologies extend periodontal research beyond predefined targets by simultaneously characterizing microbial communities, gene expression, proteins, metabolites, and regulatory mechanisms. These layers address different biological questions and should not be treated as equivalent diagnostic tests. Microbiome profiles describe ecological composition and functional potential; transcriptomic and proteomic profiles reflect dynamic biological responses; genomic markers primarily address inherited susceptibility; and epigenetic signals may reflect both susceptibility and consequences of inflammation. Association with established disease, cross-sectional discrimination, prediction of progression, and improvement of clinical decisions are therefore distinct outcomes requiring different study designs [9,13,27].

Broad molecular coverage facilitates discovery of coordinated host–microbiome patterns but also increases false-positive findings and overfitting, particularly when thousands of features are evaluated in small samples. A signature derived from established cases and healthy controls may explain biological differences or classify those groups without identifying active attachment loss, anticipating progression, or improving treatment selection. Clinical translation requires a prespecified intended use, clinically representative cohorts, external validation, calibration, and comparison with conventional clinical and radiographic models [9,28].

#### 3.5.1. Microbiome Sequencing and Functional Profiling

16S rRNA gene sequencing enables culture-independent assessment of bacterial community composition, including difficult-to-cultivate taxa, but generally provides relative abundance estimates and limited strain-level resolution. Shotgun metagenomics offers greater taxonomic resolution and identifies microbial genes and functional potential, although the presence of a gene does not demonstrate its expression. Metatranscriptomics more directly evaluates transcriptional activity but is more sensitive to RNA degradation, sequencing depth, normalization, and low-abundance signals [13]. These methods provide progressively richer biological information, but greater complexity does not automatically yield greater clinical validity.

Geng et al. analyzed eight shotgun-metagenomic cohorts from five countries and identified 54 species and 26 metabolic pathways consistently altered in at least three cohorts. Taxonomy-based classifiers achieved mean internal and external AUCs of 0.86 and 0.85, respectively, supporting a reproducible community-level signature across geographically distinct datasets. Nevertheless, the models discriminated previously diagnosed cases from controls and were not designed to identify current attachment loss or predict future progression. Functional classifiers performed less consistently, and combining taxonomic and functional profiles did not improve performance over taxonomy alone [29].

Similarly, a meta-analysis reported favorable cross-sectional discrimination for selected taxa and microbial combinations but substantial heterogeneity in sampling, case definitions, analytical methods, and thresholds [23]. Current sequencing evidence therefore supports the microbiome as a community-level correlate of periodontal status, not as a validated standalone marker of activity or progression.

#### 3.5.2. Transcriptomic and Proteomic Host-Response Signatures

Transcriptomics can identify pathways related to inflammation, immune-cell activation, extracellular-matrix remodeling, and tissue destruction. A meta-analysis of two gingival RNA-sequencing datasets identified 78 differentially expressed genes and several affected pathways, but the results were derived from secondary analysis and were not tested as a locked diagnostic model in an independent cohort [30]. Bulk-tissue signals may also reflect different proportions of epithelial, stromal, and immune cells rather than disease-specific regulation within individual cell types. Single-cell approaches can refine cellular resolution, although periodontal studies remain predominantly mechanistic and exploratory [31].

Proteomics provides more proximal information on inflammatory and tissue-remodeling processes. In 190 participants recruited at two centers, Grant et al. developed a panel containing MMP-9, S100A8, alpha-1-acid glycoprotein, pyruvate kinase, and age. It distinguished health or gingivitis from periodontitis with an AUC of 0.970, but performance was lower for health versus gingivitis and for mild-to-moderate versus advanced periodontitis. The model used leave-one-out cross-validation and data-derived thresholds without final validation in an independent external population, and it was not designed to predict progression or response to therapy [32].

Evidence synthesis indicates that additional biomarkers do not necessarily improve performance. Salivary pairs containing IL-1β, IL-6, and MMP-8 achieved favorable median sensitivity and specificity, whereas combinations of three or four markers did not substantially outperform the strongest two-marker models [33]. Untargeted proteomic studies have also produced widely variable numbers of candidate proteins and infrequently report diagnostic-accuracy measures; S100A8, the only marker quantitatively synthesized in one review, achieved an AUC of 0.71 [34]. These findings support proteomics for candidate discovery but reveal limited reproducibility and external validation. No host biomarker or panel has yet demonstrated sufficient evidence for routine diagnostic or prognostic implementation [28].

#### 3.5.3. Genomic, Epigenomic, and miRNA Susceptibility Markers

Germline variants are stable markers of inherited susceptibility, not indicators of current periodontal activity. A systematic review of 607 genetic studies found that only two directly reported diagnostic performance, identified no chairside genetic tests, and found no marker with sufficient clinical value for distinguishing health from disease or predicting progression or resolution. Genomic information may ultimately contribute to polygenic risk assessment or evaluation of rare early-onset conditions, but it is not currently a diagnostic test for common periodontitis [27].

Epigenetic features and microRNAs (miRNAs) are potentially dynamic because they reflect interactions among genetic background, environmental exposures, cellular composition, and inflammation. This plasticity is also a limitation: differences observed in established periodontitis may be consequences rather than causes of disease. Gingival-tissue studies have identified differential expression of selected miRNAs, but small samples, tissue-based collection, absence of diagnostic thresholds, and lack of longitudinal or external validation prevent direct extrapolation to non-invasive clinical tests [35].

A meta-analysis estimated an AUC of approximately 0.78 for gingival crevicular fluid miRNA profiles, but heterogeneity was very high and few studies could be combined [28]. 

DNA-methylation findings are similarly preliminary. In a study comparing 20 healthy participants with 20 treated, clinically stable patients, an initial difference in LINE-1 methylation was no longer significant after adjustment for age and sex, and selected inflammatory loci did not differ significantly between groups [36]. These results do not demonstrate that therapy normalizes an epigenetic signature or that the evaluated markers predict recurrence. Standardized specimens, normalization controls, prespecified thresholds, and longitudinal external validation remain necessary.

#### 3.5.4. Multi-Omics Integration and Clinical Translation

Multi-omics integration is biologically attractive because periodontitis reflects interactions among microbial ecology, host susceptibility, inflammation, and tissue metabolism. However, combining layers increases dimensionality, analytical flexibility, cost, and missing-data problems and does not inherently improve prediction. In an experimental-gingivitis model, coordinated microbial, metabolic, and cytokine changes appeared within 24–72 h after interruption of oral hygiene, showing that molecular alterations can precede overt gingival signs under controlled conditions. The model involved reversible gingival inflammation and did not establish prediction of periodontitis or attachment loss [37].

In a study integrating shotgun metagenomics and nuclear magnetic resonance metabolomics in 25 patients with Stage III–IV periodontitis and 25 controls, microbial and metabolic profiles partly shifted toward health-associated patterns after nonsurgical therapy. The single-center sample, short follow-up, absence of repeated control samples, and lack of an externally validated model limit diagnostic and prognostic interpretation [38]. The longitudinal model reported by Nagarajan et al. similarly supports integration of microbial and host markers but remains internally evaluated and high-dimensional relative to the number of progression events [26].

The principal contribution of omics is therefore biological resolution and candidate discovery rather than current clinical diagnosis. Translation requires standardization of case definitions, specimens, collection sites, laboratory platforms, bioinformatic pipelines, normalization, and outcome reporting. Smoking, diabetes, medication, treatment history, age, oral hygiene, and population structure must be considered because they can influence molecular profiles. Models should use representative populations, prespecified features and thresholds, independent geographic and temporal validation, calibration, decision-curve analysis, and direct comparison with clinical-only models. Longitudinal studies must distinguish incident disease, site-level progression, recurrence during supportive care, and treatment response.

At present, no microbiome, host-derived, genomic, epigenomic, or multi-omics signature has demonstrated sufficient performance and clinical utility to replace clinical and radiographic assessment, assign stage or grade, or reliably predict progression or treatment success [9]. The most credible translational pathway is not unlimited molecular expansion but externally validated, parsimonious models that add clinically meaningful information to established predictors and demonstrate improved decisions or patient outcomes. Table 1 summarizes the representative evidence, current clinical status, and principal limitations of the major omics approaches.

## 4. Advanced Digital and Imaging-Based Periodontal Diagnosis

Digital technologies provide new methods for acquiring, quantifying, and integrating periodontal information, but they do not modify the basis of periodontal diagnosis. Clinical examination remains essential for identifying inflammation and attachment loss, while two-dimensional radiography remains the standard imaging method for evaluating the structural consequences of periodontitis. CBCT, intraoral scanning, and artificial intelligence (AI) should therefore be considered complementary technologies whose value depends on whether they provide clinically relevant information beyond conventional examination [9,11].

A critical distinction must be made between technical performance and clinical validity. A digital system may accurately segment a structure or classify an image without demonstrating that it identifies active disease, predicts future attachment loss, improves treatment selection, or benefits patient outcomes. Its clinical relevance should therefore be assessed according to the intended use, reference standard, external validation, reproducibility, and incremental value over established methods [9,40].

### 4.1. From Conventional Imaging to Digital Periodontology

Digital periodontology encompasses three related functions: acquisition of anatomical information, automated analysis, and integration with clinical or biological variables. These functions represent different levels of evidence. Digital acquisition may improve measurement consistency, whereas automated classification requires validation against an appropriate clinical reference standard. Predictive models additionally require longitudinal evaluation in independent populations.

Current evidence is strongest for AI-assisted interpretation of routinely acquired two-dimensional radiographs. Evidence supporting digital prediction of periodontal deterioration remains less developed. A recent review identified 40 studies evaluating digital technologies for screening or diagnosis but only eight investigating prognosis. All eight prognostic studies were considered at high risk of bias, and their models included between 6 and 74 predictors, indicating considerable methodological heterogeneity. The evidence was therefore considered promising but inconsistent and inconclusive [40]. Digitalization may reduce observer variability, but it cannot compensate for inadequate reference standards, unrepresentative samples, or absent external validation.

### 4.2. Cone-Beam Computed Tomography and Volumetric Analysis

CBCT provides three-dimensional visualization of periodontal hard tissues without the superimposition and projection distortion inherent to conventional radiography. It can improve characterization of buccal and lingual bone plates, intrabony defects, and furcation involvement. Nevertheless, CBCT should not be considered the general reference standard for periodontal diagnosis. Two-dimensional radiographs remain the standard approach, whereas CBCT is recommended selectively when the additional information is expected to influence treatment planning and justify the radiation exposure and cost [11,39].

The strongest quantitative evidence concerns furcation involvement in maxillary molars. A systematic review and meta-analysis reported pooled sensitivity and specificity of 0.98 and an accuracy of 0.99 for CBCT compared with intra-surgical measurements. However, these estimates were derived from only three sufficiently homogeneous studies involving 168 maxillary first and second molars and cannot be generalized to all periodontal defects. Evidence for crestal bone-loss assessment was more variable, with sensitivity ranging from 0.21 to 1.00 and specificity from 0.69 to 1.00 [9,41].

**Figure 1:**
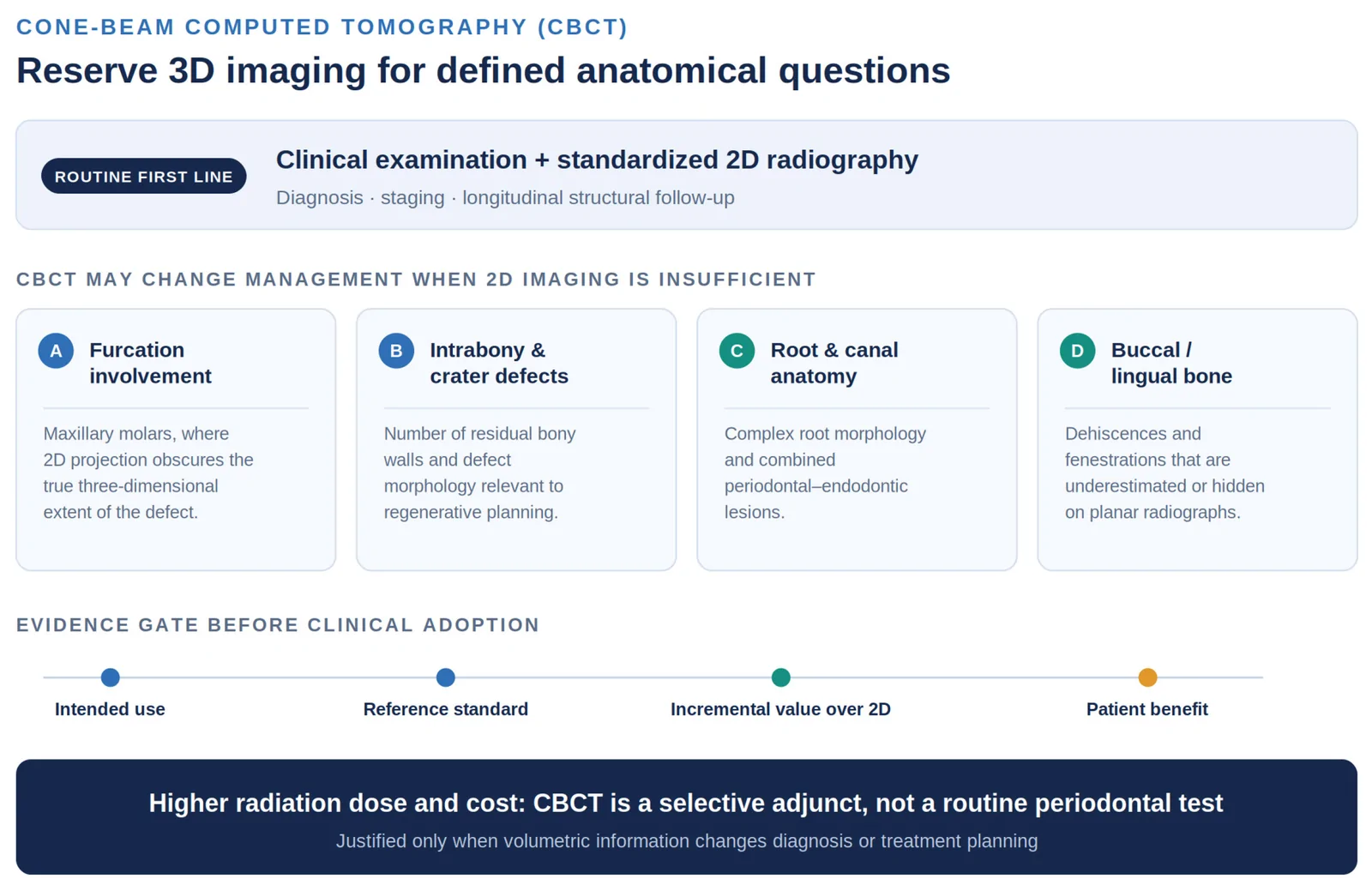
Clinical Indications and Translational Requirements of CBCT in Periodontal Diagnosis.

Volumetric reconstruction and semi-automated segmentation may quantify defect dimensions and support surgical planning. However, measurements depend on acquisition parameters, field of view, metallic artifacts, segmentation thresholds, software, and examiner correction. Standardized protocols for interpreting longitudinal volumetric changes as evidence of stability or progression remain unavailable [11]. Moreover, CBCT demonstrates accumulated anatomical destruction rather than current inflammatory activity. Repeated acquisition solely for routine periodontal monitoring is therefore difficult to justify without evidence that it changes clinical management. An illustration of these concepts can be found in Figure 1.

### 4.3. Intraoral Scanning and Three-Dimensional Surface Analysis

Intraoral scanners provide radiation-free three-dimensional records of dental and visible soft-tissue surfaces. Their main periodontal advantage is the ability to store digital models and superimpose serial scans to quantify changes in gingival recession and tissue contours. A scoping review of 52 studies identified promising applications for evaluating gingival thickness—particularly when scanning was combined with CBCT—and keratinized tissue dimensions. In contrast, plaque detection and digital estimation of probing depth showed limited success and were affected by examination bias and difficulties in visualizing posterior and proximal areas [42].

Intraoral scanning should therefore be considered a method for documenting surface morphology rather than a validated replacement for periodontal probing. It does not directly measure clinical attachment loss, subgingival inflammation, or pocket morphology. Results may also be influenced by tissue displacement, moisture, scanning trajectory, landmark visibility, registration procedures, and analytical software. Heterogeneity among scanners and protocols further limits comparability and clinical standardization [42].

Registration of intraoral surface files with CBCT data can generate combined representations of the dentogingival complex and assist anatomical visualization or surgical planning. Nevertheless, technical co-registration does not demonstrate improved diagnostic discrimination or prediction of progression. Its clinical value depends on whether the integrated model changes management beyond the separate clinical, radiographic, and surface assessments.

### 4.4. Artificial Intelligence in Periodontal Imaging

AI models can automate tooth localization, identification of anatomical landmarks, estimation of radiographic bone levels, and classification of bone-loss severity. Early studies demonstrated feasibility but predominantly used retrospective datasets and internal validation. Chang et al. analyzed standardized full-mouth radiographs from 236 patients and reported an average accuracy of 0.87 ± 0.01 for distinguishing bone loss below 15% from bone loss of 15% or greater using fivefold cross-validation. However, this binary threshold did not reproduce the complete clinical diagnosis or staging of periodontitis, and no independent external cohort was included [43].

Performance has not been uniformly high. In 595 bitewing radiographs, an AI model developed to identify landmarks and measure radiographic bone loss achieved an F1 score of 66.89%, precision of 61.1%, sensitivity of 73.9%, and mean average precision of 73.8%. The automated measurements were not directly compared with manual measurements by periodontal specialists, and further validation was considered necessary [44]. This illustrates how performance depends on the task, dataset, annotation procedure, and outcome definition.

More robust evidence was provided by a multicenter study of HC-Net+, pretrained and fine-tuned using 10,881 panoramic radiographs. The model was tested in 382 clinically labeled radiographs representing 10,198 teeth and in 760 radiographs from four international centers classified by a specialist panel. It achieved an AUROC of 94.2%, compared with 85.6% for periodontal specialists, in the clinically referenced dataset. In the multicenter radiographically referenced dataset, it achieved an AUROC of 0.967, sensitivity of 0.956, specificity of 0.826, and 92.4% correct classification at the selected threshold [45].

The study represents an important advance because it incorporated external testing and clinical reference data. Nevertheless, the model separated stages II–IV from periodontal health, gingivitis, and stage I periodontitis. It could not identify stage I and showed greater difficulty with localized stage II disease. Furthermore, the second external dataset used specialist interpretation of panoramic radiographs rather than a full-mouth clinical examination as the reference. The findings therefore support AI-assisted screening for established periodontitis but not independent diagnosis of early disease, inflammatory activity, or future progression [45].

Systematic reviews also indicate variable methodological quality and generalizability. Patil et al. included only six eligible studies using heterogeneous algorithms and radiographic modalities, preventing firm conclusions regarding clinical superiority [46]. Farina et al. similarly concluded that AI-assisted analysis of two-dimensional radiographs is among the most promising applications, although the overall evidence remains inconsistent [40].

AI-based CBCT analysis may facilitate segmentation and three-dimensional characterization, but it is less clinically validated than AI analysis of two-dimensional radiographs. Performance remains task- and dataset-dependent, and the availability of an algorithm does not justify CBCT acquisition; the indication for ionizing imaging must be established independently.

### 4.5. Artificial Intelligence-Assisted Thermal Imaging

Infrared thermography has been proposed as a non-contact method for evaluating temperature differences associated with gingival inflammation. In an exploratory study of 40 participants, 160 thermal images generated 1,734 data points labeled principally according to bleeding on probing. An XGBoost model evaluated through fivefold cross-validation achieved 92.74% accuracy, 92.95% precision, 92.74% sensitivity, and an F1 score of 92.78% [47].

These findings demonstrate technical feasibility but do not validate thermography for diagnosing periodontitis. The reference outcome was gingival inflammation rather than attachment loss, bone loss, or progression, and the observations were derived from only 40 participants rather than independent clinical cases. The absence of external validation and the potential influence of breathing, environmental conditions, vascular variation, and camera calibration further limit generalizability. AI-assisted thermography should therefore remain classified as an experimental method for evaluating gingival inflammatory patterns.

### 4.6. Multimodal Integration and Digital Patient Models

Integration of clinical records, radiographic images, intraoral scans, photographs, and automated analytical models may provide a more structured digital representation of the periodontal patient. Registration of DICOM and surface-scan files can combine hard- and soft-tissue anatomy, while AI may facilitate segmentation and measurement. However, an integrated anatomical model should not automatically be described as a periodontal “digital twin.” A true digital twin would require continuous updating and validated simulation of patient-specific disease trajectories. Current evidence mainly supports static or serial digital representations rather than predictive systems [11,40,42].

The clinical value of multimodal integration depends on demonstrating benefit beyond conventional examination. Models should be compared with clinical-only and radiographic-only approaches and evaluated using patient-level separation of training and testing data, independent multicenter validation, calibration, prespecified thresholds, and assessment of their influence on clinical decisions. Interoperability, population representativeness, privacy, transparency, cost, and automation bias must also be addressed [9,40].

**Figure 2:**
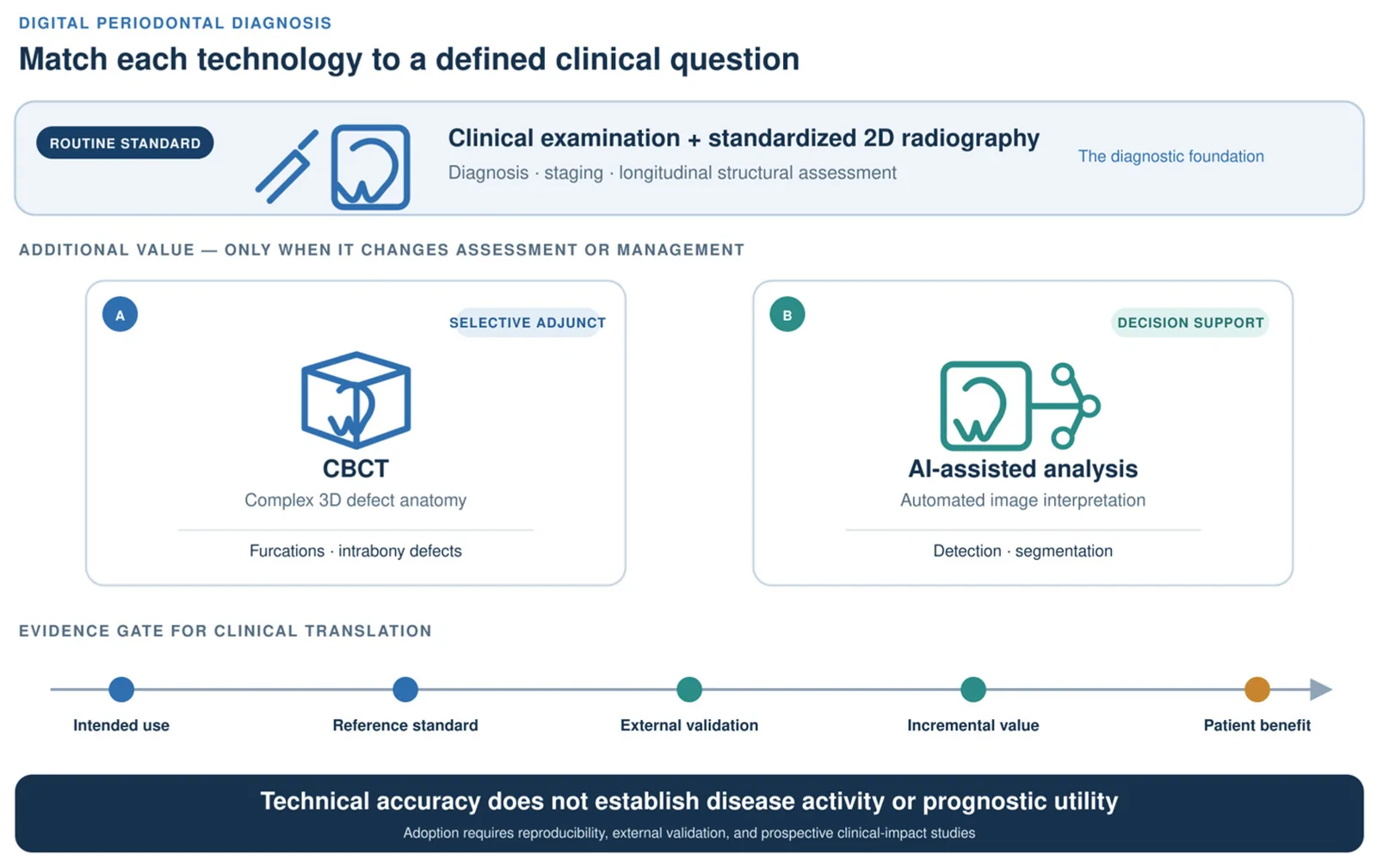
Clinical Roles and Translational Requirements of Digital Imaging Tools in Periodontal Diagnosis.

The most clinically advanced application is AI-assisted interpretation of routinely indicated two-dimensional radiographs, particularly for screening and quantifying established bone loss. CBCT is valuable for selected complex defects but is not justified for routine diagnosis. Intraoral scanning is useful for monitoring external soft-tissue morphology but cannot replace probing. AI-assisted thermography and periodontal digital-twin models remain experimental. Digital technologies should therefore be adopted according to demonstrated added clinical value rather than technical sophistication alone. An illustration of these concepts can be found in Figure 2.

Clinical examination and standardized two-dimensional (2D) radiography constitute the diagnostic foundation for periodontitis. Cone-beam computed tomography (CBCT) may provide complementary three-dimensional anatomical information in selected cases, particularly for evaluating furcation involvement and intrabony defects. Artificial intelligence (AI)-assisted image analysis may support detection and segmentation but should be regarded as decision support rather than an independent diagnostic method. Clinical translation requires a clearly defined intended use, comparison with an appropriate reference standard, external validation, demonstration of incremental value beyond conventional assessment, and evidence of patient benefit. Technical accuracy alone does not establish disease activity, prognostic validity, or clinical utility.

## 5. Biological Fluids and Periodontal Biomarkers

Biological fluids provide access to host- and microbiome-derived signals that are not captured directly by clinical probing or radiographic examination. Saliva, gingival crevicular fluid (GCF), and serum differ substantially in their biological origin and diagnostic scope. Saliva represents a whole-mouth mixture influenced by multiple oral sites; GCF provides localized information from an individual sulcus or periodontal pocket; and serum predominantly reflects systemic rather than periodontal inflammation. These matrices should therefore not be considered interchangeable.

Although numerous biomarkers distinguish patients with periodontitis from healthy controls, this cross-sectional discrimination does not necessarily demonstrate the ability to identify active attachment loss or predict future progression. Clinical translation requires predefined thresholds, reproducible sampling, independent external validation, and evidence that biomarker testing adds useful information to conventional periodontal examination. The distinction between association, diagnostic discrimination, longitudinal prediction, and treatment monitoring is essential when interpreting the available evidence [28].

### 5.1. Saliva as a Diagnostic Matrix

Saliva is easily collected, non-invasive, and suitable for repeated or community-based sampling. It contains glandular secretions, GCF, epithelial cells, microorganisms, inflammatory mediators, enzymes, proteins, and metabolites. This composition provides a broad representation of the oral environment but prevents direct attribution of a biomarker concentration to a specific periodontal site. Salivary measurements may also be affected by flow rate, stimulation, collection time, smoking, oral bleeding, medication, and other oral or systemic inflammatory conditions.

Among the most extensively investigated host-derived salivary biomarkers are IL-1β, IL-6, MMP-8, MMP-9, calprotectin, osteoprotegerin, and proteins associated with bone metabolism. A 2025 systematic review identified 61 diagnostic-accuracy studies, of which only 13 could be included in meta-analyses distinguishing periodontal health from periodontitis. Grouped salivary bone markers showed an AUC of 0.91, compared with 0.86 for cytokines and 0.77 for matrix metalloproteinases. However, performance varied across markers, case definitions, analytical platforms, and thresholds. Moreover, combining cytokines and metalloproteinases did not produce a statistically significant improvement when distinguishing periodontitis from gingivitis, and evidence concerning progression or disease resolution was insufficient for quantitative synthesis [28].

Multivariable protein panels may capture a broader biological response than isolated markers. In a two-centre study of 190 participants, a model combining MMP-9, S100A8, alpha-1-acid glycoprotein, pyruvate kinase, and age achieved an AUC of 0.970 for distinguishing periodontitis from health or gingivitis. The same approach produced lower AUCs for distinguishing health from gingivitis and mild-to-moderate from advanced periodontitis. Model performance was estimated using leave-one-out cross-validation, and age contributed to the best-performing periodontitis model. Consequently, the findings demonstrate internal discriminatory capacity but do not constitute independent prospective validation or establish prediction of future progression [32].

More recent untargeted proteomic research similarly illustrates the difference between discovery and clinical validation. Blanco-Pintos et al. evaluated saliva from 44 periodontally healthy participants and 41 patients with periodontitis. Eight individual proteins produced bias-corrected accuracies ranging from 78.8% to 86.8%. Adjustment for age increased the estimated accuracy to 94.1–98.2%, whereas adjustment for smoking produced smaller improvements. These findings suggest that demographic characteristics can strongly influence model performance. However, the small sample, internal bias correction, multiple candidate proteins, and absence of an independent external cohort preclude immediate clinical application [33].

Longitudinal evidence provides a more relevant assessment of potential disease activity. Teles et al. monitored 113 periodontally healthy participants and 302 patients with periodontitis every two months for one year. Participants with periodontitis who developed progression at three or more sites showed persistently higher salivary levels of IFN-γ, IL-6, VEGF, IL-1β, MMP-8, IL-10, and OPG. Non-surgical periodontal therapy was followed by clinical improvement and reductions in several salivary analytes [48]. This study supports a relationship between sustained salivary inflammatory profiles and progression. Nevertheless, it did not establish a clinically applicable threshold or externally validate a predictive model. Elevated biomarker levels should therefore be interpreted as longitudinal associations rather than as a validated test capable of prospectively identifying individual progressors.

Microbial salivary biomarkers are addressed in the microbiological and omics sections of this review. Their presence in saliva may support the characterization of dysbiosis but does not independently establish microbial viability, site-specific destruction, or future attachment loss.

### 5.2. Active MMP-8 Point-of-Care Testing

Active matrix metalloproteinase-8 is a neutrophil-derived collagenase associated with extracellular-matrix degradation. Oral-rinse point-of-care tests were developed to translate this biomarker into a rapid chairside or non-dental screening method. Their clinical performance, however, is more moderate than suggested by early individual studies.

Two independent diagnostic studies conducted in Hong Kong and Shanghai included 384 and 390 participants, respectively. In these populations, a positive aMMP-8 oral-rinse test produced AUROCs of 0.661 and 0.669. An updated meta-analysis combining eight studies and 2,048 participants estimated a sensitivity of 0.59, specificity of 0.82, and hierarchical summary AUROC of 0.77, with moderate certainty of evidence [49].

The moderate-to-high specificity suggests that a positive result may support referral or additional periodontal assessment, particularly in non-dental settings. However, the sensitivity indicates that approximately four of every ten affected individuals may receive a negative result. The test therefore cannot replace full-mouth periodontal examination. It may have a role as an adjunctive screening component or within a multivariable diagnostic strategy, but its utility for monitoring treatment, predicting attachment loss, or improving clinical outcomes requires further prospective validation [49].

### 5.3. Gingival Crevicular Fluid

GCF is derived from the gingival sulcus or periodontal pocket and contains plasma proteins, inflammatory mediators, immune-cell products, microbial components, and enzymes released during tissue remodeling. Its principal advantage is site specificity: a sample can be anatomically related to a particular periodontal site. This characteristic is valuable for investigating local biological activity but makes collection and interpretation more technically demanding than salivary analysis.

The strongest recent longitudinal evidence comes from a study in which 112 periodontally healthy participants and 302 patients with periodontitis were monitored every two months for one year. Sixty-four GCF biomarkers were measured at progressing and stable sites. Progressing sites showed significantly higher concentrations of IL-1β, MMP-8, IL-12p40, epidermal growth factor, and vascular endothelial growth factor. In contrast, MMP-9 and periostin were more elevated at stable sites. Non-surgical periodontal treatment produced reductions in several inflammatory mediators and changes in the GCF profile [48].

These findings demonstrate that progressing and stable sites may have different molecular patterns and that these profiles respond to treatment. Nevertheless, the study compared biomarker trajectories between groups and sites rather than validating a prespecified diagnostic test. It did not establish universal cut-off values, external predictive performance, or incremental value over baseline probing depth, attachment level, and bleeding on probing. The results therefore support biological plausibility and biomarker development but not routine diagnosis based on GCF alone.

Evidence for other GCF markers remains more limited. In the meta-analysis by Rakic et al., GCF microRNAs were the only GCF biomarker category that could be quantitatively synthesized and produced an AUC of 0.79. The small number of studies and incomplete reporting prevented a comprehensive assessment of clinical utility [28].

GCF sampling is also susceptible to contamination with blood or saliva, variation in collection duration and sampled volume, site selection, paper-strip positioning, and differences in laboratory normalization. Because multiple periodontal sites may behave differently within the same patient, results from one or a few sites may not represent the patient-level condition. These factors currently restrict GCF biomarkers primarily to research and selected monitoring applications.

### 5.4. Systemic Biomarkers

Periodontitis is associated with systemic inflammation, and circulating markers such as C-reactive protein (CRP), high-sensitivity CRP, IL-6, MMP-8, MMP-9, and myeloperoxidase have been investigated in serum. A systematic review and meta-analysis found that patients with periodontitis had higher CRP and high-sensitivity CRP concentrations than periodontally healthy controls. Non-intensive periodontal therapy was followed by a progressive reduction in high-sensitivity CRP over six months, although heterogeneity among studies was substantial [50,51].

These findings support an association between periodontitis and systemic inflammatory burden but do not establish serum CRP as a periodontitis-specific diagnostic test. CRP is influenced by infections, obesity, smoking, cardiovascular and metabolic diseases, medication, and other inflammatory conditions. Elevated concentrations therefore cannot be attributed to periodontal inflammation without considering the broader medical context.

This limitation was demonstrated in the longitudinal study by Teles et al. Serum analytes were not associated with periodontal progression, although non-surgical treatment reduced serum CRP, MMP-8, MMP-9, and myeloperoxidase [48]. Serum biomarkers may therefore be relevant for investigating the systemic inflammatory effects of periodontitis and treatment, but current evidence does not support their use for site-specific diagnosis, staging, grading, or prediction of periodontal progression.

### 5.5. Clinical Applications, Advantages, and Limitations

The potential value of biological-fluid testing depends on the clinical question. Saliva is the most practical matrix for population screening and repeated sampling but represents a pooled whole-mouth signal. GCF offers greater site specificity but requires technically standardized collection and does not readily provide a patient-level diagnosis. Serum reflects systemic inflammatory burden but lacks periodontal specificity. Selection of a matrix should therefore be based on the intended diagnostic use rather than on biomarker availability alone (Table 2).

The current evidence is strongest for discriminating established periodontitis from periodontal health. Evidence for differentiating gingivitis from early periodontitis, prospectively predicting attachment loss, identifying recurrence, and directing treatment remains substantially weaker. High internal AUCs reported by exploratory studies should not be interpreted as evidence of clinical readiness when thresholds are derived within the same population or when age and other strong clinical predictors are incorporated without comparison against a clinical-only model.

**Figure 3:**
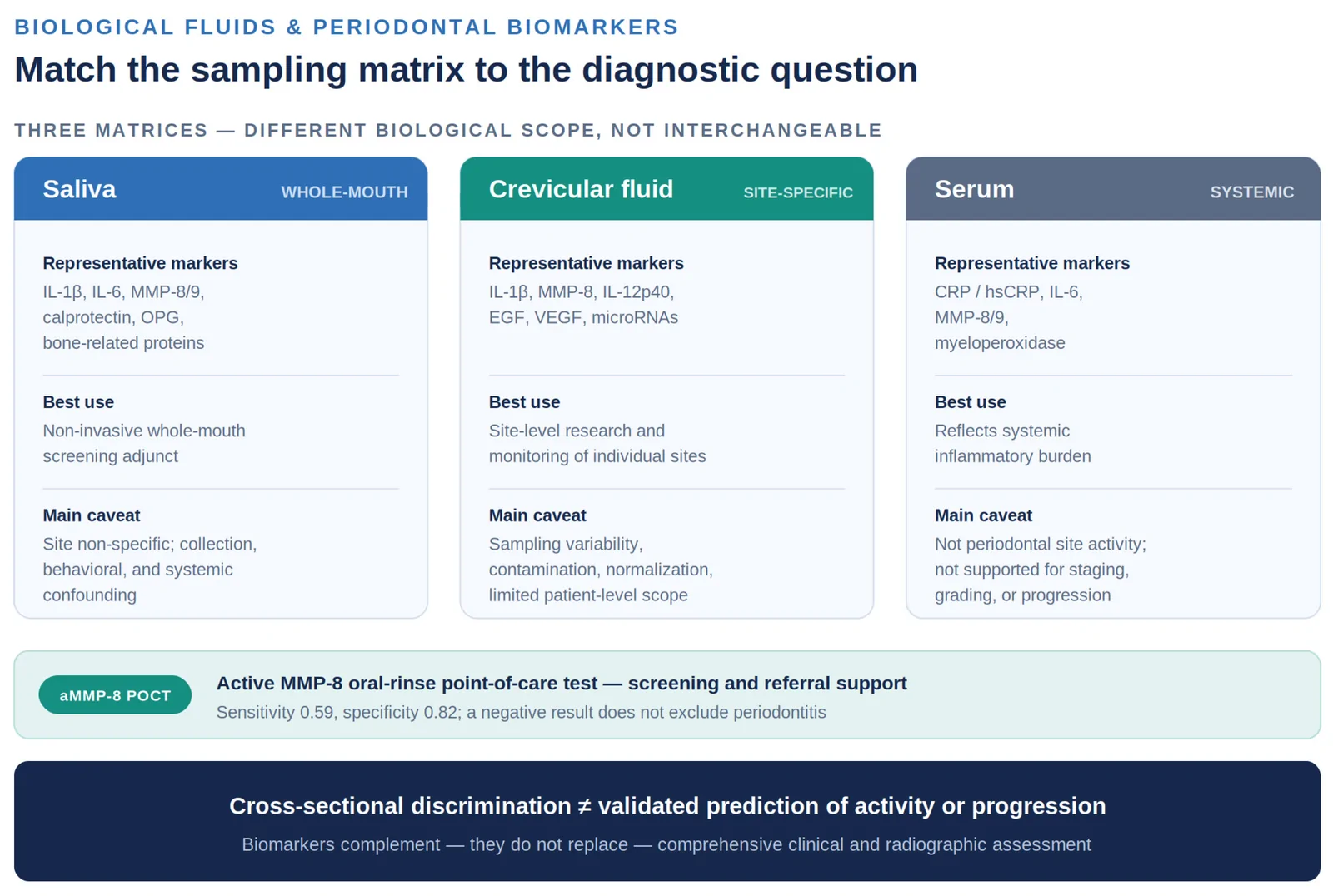
Biological Fluids and Biomarkers in Periodontal Assessment: Scope, Representative Markers, and Interpretation.

Future studies should use prospective multicentre designs, standardized case definitions and collection protocols, prespecified thresholds, blinded assessment, and independent external validation. Biomarker models should be compared directly with conventional examination and evaluated for calibration, incremental diagnostic value, cost-effectiveness, and their capacity to change clinical decisions or improve patient outcomes. Until these requirements are met, biological-fluid biomarkers should remain adjuncts to clinical and radiographic assessment rather than a standalone diagnostic test. An illustration of these concepts can be found in Figure 3.

## 6. Predictive Models and Machine Learning in Periodontal Diagnosis

### 6.1. From Descriptive Assessment to Predictive Periodontology

Artificial intelligence applications in periodontology are related with cross-sectional disease classification, automated radiographic assessment, and prediction of future clinical outcomes. These should not be considered equivalent. A model that distinguishes established periodontitis from periodontal health may support diagnosis or screening but does not necessarily identify active disease or predict future attachment loss.

Classical statistical methods, such as logistic regression and Cox proportional hazards models, remain important because they provide relatively transparent and interpretable estimates. Machine learning algorithms—including Random Forest, Support Vector Machines, gradient boosting, and neural networks—can model nonlinear relationships among clinical, demographic, molecular, microbial, and imaging variables. Deep learning, particularly convolutional neural networks, has been applied mainly to radiographic segmentation and classification. Nevertheless, increasing algorithmic complexity does not automatically improve generalizability.

Bashir et al. trained ten machine learning algorithms using national survey datasets from Taiwan (*n* = 3453) and the United States (*n* = 3685). Although several models achieved internal AUCs and accuracies exceeding 0.95, their performance declined markedly when validated using data from the alternative country. Furthermore, the outcome was existing periodontitis rather than prospective progression. This finding demonstrates that excellent internal performance may reflect population-specific characteristics and should not be interpreted as evidence of transportability or prognostic validity [52].

### 6.2. Automated Diagnosis and Radiographic Assessment

Radiographic bone-loss detection currently represents the most extensively investigated application of deep learning in periodontology. A systematic review of 30 studies, including 10 eligible for meta-analysis, reported pooled sensitivity of 87%, specificity of 76%, and accuracy of 84% for machine- and deep-learning models applied to panoramic and periapical radiographs. However, most studies were classified as having intermediate methodological quality, none reached the highest APPRAISE-AI quality category, and important heterogeneity was found in datasets, reference standards, validation procedures, and reporting [53].

More recently, Xue et al. developed an ensemble model combining YOLOv8, Mask R-CNN, and TransUNet for tooth detection, tissue segmentation, quantification of periodontal bone loss, and radiographic estimation of periodontitis stage. The study included panoramic radiographs from 320 patients and 8462 teeth. Compared with annotations produced by three periodontists, the model showed a bone-loss measurement deviation of 5.28%, a Pearson correlation coefficient of 0.832, an intraclass correlation coefficient of 0.806, and an overall diagnostic accuracy of 89.45% [54].

These findings demonstrate the feasibility of automating radiographic measurements and severity classification. Nevertheless, the model was evaluated within a single dataset and did not provide independent multicenter external validation. In addition, radiographic bone loss represents only one component of periodontal staging. Complete stage and grade assignment also requires clinical attachment loss, periodontitis-related tooth loss, complexity factors, evidence of progression, and relevant risk modifiers. Therefore, the model cannot independently determine current disease activity or predict future progression.

### 6.3. Prediction of Progression and Tooth Loss

Evidence supporting genuine longitudinal prediction remains more limited. Furquim et al. analyzed data from 415 participants—113 periodontally healthy individuals and 302 patients with periodontitis—who were monitored every two months for one year. The best-performing probabilistic graphical model combined clinical measurements, salivary IL-1β, age, and sex and achieved an AUROC of 0.88, sensitivity of 0.55, and specificity of 0.81. The number of deep periodontal pockets was the most influential predictor, indicating that conventional clinical information remained central even after biomarkers were incorporated. Despite its promising discrimination, the model still requires independent external validation, assessment of calibration, and evaluation of clinical benefit [55].

Stronger external-validation evidence is available for tooth-loss prognosis. Troiano et al. analyzed 515 patients and 3157 molars from four centers located on different continents. An ensemble combining a neural network and logistic regression achieved an externally validated AUC of 0.726 for overall 10-year molar loss, while the neural network achieved an AUC of 0.724 for periodontitis-related molar loss. Although these values were more moderate than many internally validated results, they provide a more realistic estimate of model performance across populations. However, this model predicts a tooth-level outcome after periodontal treatment and should not be interpreted as predicting periodontitis onset or real-time disease activity [56].

### 6.4. Challenges and Future Development Strategies

The principal challenge is not developing increasingly complex algorithms but demonstrating that their predictions remain accurate, calibrated, and clinically useful outside the development population. Future studies should employ multicenter and temporally separated external validation, patient-level data partitioning, standardized periodontal definitions, transparent management of missing data and class imbalance, and direct comparison with prespecified clinical-only models. Model evaluation should include calibration and clinical decision benefit in addition to AUC, sensitivity, specificity, and accuracy.

Multimodal models integrating clinical examination, radiographic measurements, biomarkers, microbiome profiles, and electronic health records may improve biological resolution. However, additional data layers can also increase dimensionality, overfitting, cost, and missing-data problems. Their inclusion should therefore be justified by demonstrating incremental value over conventional periodontal assessment. Explainability, population fairness, reproducibility, data protection, and interoperability must also be considered before clinical adoption [57,58].

At present, artificial intelligence is best positioned as a decision-support tool for standardizing measurements, assisting screening, and complementing clinical risk assessment. Its ability to independently identify active disease or predict progression and therapeutic response remains insufficiently validated for routine periodontal practice.

## 7. Therapeutic Implications of Advanced Periodontal Diagnosis: Diagnostic Evidence as a Framework for Treatment Selection

Advanced diagnostic information is clinically valuable only when it changes treatment selection or monitoring and ultimately improves patient outcomes. Current evidence is asymmetrical: clinical and radiographic findings already guide treatment complexity and regenerative decisions, whereas biomarkers, omics profiles, and AI remain predominantly diagnostic or prognostic tools without validated therapeutic thresholds.

### 7.1. Diagnostic Evidence as a Framework for Treatment Selection

The EFP clinical practice guidelines organize periodontal treatment as a stepwise process comprising behavioral and risk-factor control, supra- and subgingival biofilm management, treatment of residual pockets, and supportive periodontal care. Stage IV additionally identifies functional and rehabilitative complexity requiring interdisciplinary management [9,59]. Within this framework, staging informs the anatomical demands of treatment, while grading and modifiers such as smoking and diabetes influence risk control and monitoring intensity.

However, neither staging and grading nor currently available biomarkers constitute prescriptive algorithms for selecting host-modulatory drugs, regenerative biomaterials, or systemic antimicrobials. Although molecular markers may provide information about inflammatory burden, the literature has not established prospectively validated thresholds demonstrating that biomarker-guided allocation improves clinical attachment, tooth retention, or other patient-relevant outcomes [33,59]. Contemporary diagnostic personalization therefore remains primarily risk-adapted rather than molecularly prescribed.

### 7.2. Host- and Microbiome-Directed Adjuncts: Biological Plausibility Versus Clinical Readiness

Mechanical disruption of the subgingival biofilm remains the therapeutic foundation because it simultaneously reduces microbial burden and downstream inflammatory signaling. Host-modulation strategies offer a biologically plausible complement, but their clinical maturity differs substantially. Subantimicrobial-dose doxycycline has the most developed mechanistic and clinical background, particularly through inhibition of matrix metalloproteinases [60]. Nevertheless, the EFP guideline did not support its routine adjunctive use because the additional clinical benefit was insufficient to justify a general recommendation. The same guideline recommended or suggested against routine adjunctive use of omega-3 fatty acids, probiotics, and locally delivered statins because of limited, heterogeneous, or poorly generalizable evidence [59].

Complement inhibition illustrates the distinction between proof of mechanism and demonstrated periodontal efficacy. In a phase IIa randomized split-mouth trial, locally administered AMY-101 reduced gingival inflammation, bleeding on probing, and GCF levels of MMP-8 and MMP-9. However, the participants predominantly presented gingivitis, changes in probing depth and clinical attachment were not meaningfully different between treatments, and efficacy as an adjunct to mechanical treatment of periodontitis was not established [61]. AMY-101 should therefore be considered a promising investigational host-modulatory strategy rather than a validated periodontitis therapy.

A similar caution applies to microbiome-directed interventions. Systemic antibiotics may be considered for selected clinical categories but are not recommended routinely, and no microbial threshold has been validated for choosing an antibiotic regimen or demonstrating that test-guided prescribing improves outcomes. Probiotics and other ecological strategies remain conceptually attractive, but evidence of durable microbiome restoration and clinically relevant attachment gains remains insufficient for routine implementation [59].

### 7.3. Regeneration and Minimally Invasive Surgery: Defect Anatomy Remains the Most Actionable Treatment-Selection Variable

Compared with molecularly guided interventions, regenerative therapy has a substantially stronger diagnostic–therapeutic connection because treatment selection is based on clinically and radiographically identifiable defect characteristics. The EFP guideline recommends regenerative surgery for residual deep pockets associated with intrabony defects of at least 3 mm [59]. A systematic review of 79 randomized trials found that regenerative procedures produced an additional mean clinical attachment gain of 1.34 mm compared with open-flap debridement. Enamel matrix derivatives and guided tissue regeneration were both superior to access-flap surgery, although heterogeneity was moderate to high and only 10 studies had a low risk of bias [62]. Medium- and long-term evidence also favors regenerative approaches over open-flap debridement, but does not establish a definitive hierarchy among regenerative materials or combinations [63].

Minimally invasive surgery should be interpreted as a tissue-preserving delivery strategy rather than an independent regenerative therapy. Favorable attachment gains, pocket reduction, and low morbidity have been reported, but only two randomized trials directly informed the comparison with conventional surgery in an earlier meta-analysis, limiting attribution of outcomes to flap design alone [64]. More recent evidence indicates that single-flap access may reduce postoperative pain and improve early wound healing, whereas the additional benefit and morbidity associated with biomaterials depend on defect morphology and the selected regenerative technology [65]. Treatment selection should therefore integrate defect depth and configuration, soft-tissue characteristics, wound stability, patient-level risk, and operator expertise rather than assuming that minimal access or biomaterial addition is universally superior.

### 7.4. Biomaterials and Advanced Delivery Systems: Established Scaffolds Versus Experimental Bio-Intelligence

A distinction is required between biomaterials supported by human clinical trials and emerging drug-delivery platforms. Enamel matrix derivatives, barrier membranes, and selected grafting materials have documented clinical utility in appropriately selected intrabony defects, although their relative advantages remain context-dependent [62,63]. In contrast, stimuli-responsive hydrogels, nanoparticles, exosome carriers, and sequential-release scaffolds are primarily supported by material-characterization and preclinical studies. These platforms may improve local retention and controlled delivery of antimicrobial, anti-inflammatory, or regenerative agents, but standardized manufacturing, degradation kinetics, safety, and comparative human effectiveness remain unresolved [66]. They should consequently be presented as future therapeutic strategies rather than clinically validated extensions of advanced diagnosis.

### 7.5. From Prediction to Therapeutic Utility

Accordingly, periodontal AI should currently be regarded as decision support rather than autonomous therapeutic guidance. Before clinical implementation, predictive models require external validation, calibration assessment, transparent reporting, clinically interpretable outputs, and prospective impact studies comparing algorithm-guided care with conventional decision-making [58].

The most defensible therapeutic framework is therefore evidence-calibrated: clinical and radiographic diagnosis currently guides established stepwise and regenerative care; molecular biomarkers and AI may refine risk estimation; and host modulation, microbiome engineering, and intelligent biomaterials remain investigational until diagnostic–treatment interactions and patient-relevant benefits are demonstrated prospectively.

## 8. Recommendations

Future studies should be organized around clearly defined clinical questions rather than technological performance alone. Each biomarker, imaging method, or predictive model should specify whether its intended use is screening, diagnosis, identification of site-level activity, prediction of patient-level progression, treatment monitoring, or therapeutic selection. Cross-sectional discrimination between periodontitis and health should not be interpreted as evidence of active disease or future progression. Novel approaches should be compared with conventional clinical and radiographic assessment and evaluated for their incremental contribution [9,15,28].

Biomarker and microbiome studies require standardized sampling, storage, analytical platforms, normalization procedures, case definitions, and prespecified thresholds. Prospective multicenter cohorts should assess reproducibility, sensitivity, specificity, calibration, and external validity across geographically and clinically diverse populations. Site-level and patient-level outcomes must also be distinguished because a biomarker associated with a progressing site may not reliably predict patient-level progression. Recent microbiome and longitudinal studies provide methodological advances, but neither cross-cohort discrimination nor longitudinal association alone establishes a clinically applicable predictive test [28,29,48].

Artificial intelligence and multimodal models should undergo patient-level data partitioning, independent external validation, calibration assessment, and direct comparison with prespecified clinical-only models. Reports should describe the management of missing data, class imbalance, population heterogeneity, and potential demographic or clinical bias. Model complexity should be justified by demonstrable incremental value; additional molecular, microbial, or imaging variables are not useful if they fail to improve discrimination, calibration, or clinical decisions. Explainability and transparent reporting are also essential [52,57,58].

Clinical utility should be demonstrated through prospective impact studies rather than inferred from diagnostic accuracy. For antimicrobial selection, host-modulatory approaches, regenerative procedures, and minimally invasive interventions, future trials should compare test-guided strategies with guideline-based care and determine whether they improve clinical attachment, tooth retention, safety, or resource use. Retrospective analyses in which microbial findings determined antibiotic allocation cannot establish that testing caused differences in treatment outcomes [24,25]. Biomarker-, omics-, or AI-guided treatment should remain investigational until therapeutic thresholds and patient-relevant benefits are prospectively validated [33,59].

Implementation should remain proportionate to evidence and clinical need. Clinical examination and standardized radiography should remain the diagnostic foundation, while molecular, microbiological, three-dimensional imaging, and AI-based methods should be introduced selectively when they are likely to change assessment or management. Future evaluation should incorporate reproducibility, external validation, incremental value, cost-effectiveness, workflow feasibility, interoperability, and equitable access. The priority is not technological complexity, but reliable information that improves clinical decisions and patient outcomes.

## 9. Conclusions

Periodontal diagnosis is expanding without displacing its established clinical foundation. Comprehensive clinical examination, staging and grading, and standardized two-dimensional radiography remain central to case definition, assessment of accumulated tissue destruction, treatment planning, and longitudinal follow-up. Cone-beam computed tomography can provide additional anatomical information in selected complex cases, while artificial intelligence may improve the consistency and efficiency of radiographic detection and measurement. Nevertheless, neither greater imaging resolution nor algorithmic accuracy independently demonstrates active periodontal destruction or future progression.

Molecular and omics-based approaches have substantially improved the biological characterization of periodontitis. Targeted microbial testing, microbiome sequencing, and host-derived biomarkers from saliva and gingival crevicular fluid can identify dysbiotic and inflammatory profiles associated with established disease. Transcriptomic, proteomic, and epigenomic analyses provide further insight into host–microbiome interactions and individual susceptibility. However, diagnostic discrimination between periodontitis and health is more consistently supported than prospective prediction of attachment loss, recurrence, or therapeutic response. The predominance of cross-sectional studies, internally validated models, methodological heterogeneity, and non-standardized thresholds currently limits the independent clinical use of these technologies.

The therapeutic implications of advanced diagnosis remain similarly unequal. Disease severity, risk modifiers, functional complexity, and defect anatomy already inform established stepwise and regenerative treatment, whereas biomarkers, microbiome profiles, omics signatures, and AI models do not yet provide validated thresholds for selecting antimicrobials, host-modulatory interventions, regenerative biomaterials, or minimally invasive procedures. Precision periodontology should therefore be understood as an evidence-calibrated framework in development rather than an established system of molecularly prescribed care. At present, advanced diagnostic technologies are best positioned as selective adjuncts that refine biological or anatomical assessment while clinical and radiographic findings continue to guide periodontal management.

## Figures and Tables

**Table 1:** Omics-based approaches in periodontal diagnosis: representative evidence, current status, and principal limitations.

Approach	Specimen and Targets	Representative Evidence	Level of Evidence	Advantages	Limitations/Disadvantages
Microbiome	Saliva or subgingival plaque; community and functional profiles	Eight shotgun-metagenomic cohorts from five countries; taxonomy classifiers reached mean internal/external AUCs of 0.86/0.85 [29].	Low: cross-sectional case–control cohorts with cross-country external validation; no prospective or outcome data	Non-invasive sampling; captures community structure and functional potential; multi-country external validation supports diagnostic discrimination	Sampling, platform, case-definition, and bioinformatic heterogeneity; no validated activity or progression threshold
Transcriptomics	Mainly gingival tissue; host-response pathways	Meta-analysis of two RNA-seq datasets identified candidate genes and pathways but did not externally validate a diagnostic model [30].	Very low: small datasets; no external validation of a diagnostic model	Direct mechanistic insight into host-response pathways; useful for candidate-biomarker discovery	Small datasets, batch and cell-composition effects, invasive tissue sampling, and absent clinical thresholds
Proteomics	Saliva and GCF; inflammatory and tissue-remodeling proteins	Protein-and-age panel achieved AUC 0.970 in 190 participants using internal cross-validation [32].	Low: single-cohort diagnostic accuracy with internal cross-validation only	Non-invasive matrices; high internal diagnostic accuracy; proximal to inflammatory and tissue-remodeling activity	Data-derived markers and thresholds, platform heterogeneity, and limited independent external validation
Epigenomics	Gingival tissue, GCF, or saliva; DNA methylation and miRNAs	GCF miRNA meta-analysis yielded AUC approximately 0.78 with high heterogeneity [28].	Very low: mainly small cross-sectional or association studies with high heterogeneity	Accessible matrices (saliva/GCF); reflects regulatory and susceptibility signals	Small, mainly cross-sectional studies; tissue specificity; no validated thresholds or longitudinal prediction
Integrative multi-omics	Plaque, saliva, serum, tissue, or stool; combined microbial and host information	Small longitudinal studies identified coordinated disease- or treatment-associated profiles but did not externally validate clinical models [26,38].	Very low: small exploratory studies; no external validation or clinical-impact data	Integrates microbial and host layers; potential to model biological and progression complexity	High dimensionality, overfitting, cost, missing data, and lack of calibration, external validation, and clinical-impact studies

Abbreviations: AUC, area under the receiver operating characteristic curve; GCF, gingival crevicular fluid; miRNA, microRNA; RNA-seq, RNA sequencing. Note: “Current status” reflects evidentiary support rather than technical availability. Current evidence does not support replacing clinical and radiographic examination with microbial, host-derived, genetic, or multi-omics testing [39].

**Table 2:** Clinical Utility and Readiness of Biological-Fluid Biomarkers in Periodontal Assessment.

Matrix or Test	Representative Biomarkers	Key Evidence	Clinical Interpretation
Saliva	IL-1β, IL-6, MMP-8/9, calprotectin, OPG, and bone-related proteins	Diagnostic AUCs were 0.91 for bone-related markers, 0.86 for cytokines, and 0.77 for matrix metalloproteinases [28]. Longitudinal associations were reported, but no clinical threshold was validated [48].	Non-invasive whole-mouth adjunct. Site nonspecificity and collection, behavioral, and systemic confounding limit individual prediction.
aMMP-8 oral-rinse POCT	Active MMP-8	Sensitivity, 0.59; specificity, 0.82; and HSROC, 0.77 across eight studies involving 2048 participants [49].	May support screening and referral. A negative result cannot exclude periodontitis, and the test does not replace full-mouth examination.
Gingival crevicular fluid	IL-1β, MMP-8, IL-12p40, EGF, VEGF, and microRNAs	Progressing and stable sites showed different profiles, but cutoffs and incremental predictive value were not validated [48] GCF microRNAs yielded an AUC of 0.79 [28].	Site-specific research and monitoring matrix. Sampling variability, contamination, normalization, and limited patient-level representativeness remain barriers.
Serum	CRP/hsCRP, IL-6, MMP-8/9, and myeloperoxidase	CRP and hsCRP were higher in periodontitis and decreased after therapy, with substantial heterogeneity [50]. Serum analytes did not predict progression [48].	Reflects systemic inflammatory burden rather than periodontal site activity. Not supported for diagnosis, staging, grading, or progression prediction.

Abbreviations: aMMP-8, active-matrix metalloproteinase-8; AUC, area under the receiver operating characteristic curve; CRP, C-reactive protein; EGF, epidermal growth factor; GCF, gingival crevicular fluid; HSROC, hierarchical summary receiver operating characteristic; hsCRP, high-sensitivity C-reactive protein; IL, interleukin; MMP, matrix metalloproteinase; OPG, osteoprotegerin; POCT, point-of-care test; VEGF, vascular endothelial growth factor. Note: Performance estimates derive from heterogeneous populations, biomarkers, and reference standards and should not be compared directly. These tests complement, but do not replace, comprehensive clinical and radiographic periodontal assessment.

## Data Availability

No new data were created or analyzed in this study. Data sharing does not apply to this article.

## Abbreviations

The following abbreviations are used in this manuscript:

AgNPs	Silver nanoparticles
AI	Artificial intelligence
AUC	Area under the curve
BOP	Bleeding on probing
CAL	Clinical attachment level
CBCT	Cone-beam computed tomography
CEJ	Cemento-enamel junction
CNNs	Convolutional neural networks
CRP	C-reactive protein
DL	Deep learning
DMARDs	Disease-modifying antirheumatic drugs
GCF	Gingival crevicular fluid
HMT	Host-modulation therapy
IL-4	Interleukin-4
IL-6	Interleukin-6
LPS	Lipopolysaccharide
MCP-1	Monocyte chemoattractant protein-1 (CCL2)
MeSH	Medical Subject Headings
ML	Machine learning
MMP(s)	Matrix metalloproteinase(s)
MMP-8	Matrix metalloproteinase-8
NSAIDs	Nonsteroidal anti-inflammatory drugs
NST	Non-surgical therapy
OPG	Osteoprotegerin
PCR	Polymerase chain reaction
PD	Probing depth
PGE2	Prostaglandin E2
PLGA	Poly(lactic-co-glycolic acid)
POC	Point-of-care
POCTs	Point-of-care tests
PSD	Polymicrobial synergy and dysbiosis
RA	Rheumatoid arthritis
RANKL	Receptor activator of nuclear factor kappa-B ligand
RNA-Seq	RNA sequencing
ROS	Reactive oxygen species
SDD	Subantimicrobial-dose doxycycline
SPMs	Specialized pro-resolving mediators
Th17	T helper 17 cells
TLR	Toll-like receptor
Tregs	Regulatory T cells
XAI	Explainable artificial intelligence
ZnO	Zinc oxide
